# Associations Between Physical Activity, Insomnia, and Cognitive Disengagement Syndrome (CDS) Among Young Adults Using the Adult Concentration Inventory (ACI)

**DOI:** 10.1177/10870547251355005

**Published:** 2025-08-29

**Authors:** Dena Sadeghi-Bahmani, Larina Eisenhut, Thorsten Mikoteit, Nico Helfenstein, Annette Beatrix Brühl, Kenneth M. Dürsteler, Serge Brand

**Affiliations:** 1 Stanford University, Department of Psychology, Stanford, CA, USA; 2 Psychiatric University Clinics Basel; Center for Affective, Stress and Sleep Disorders, Basel, Switzerland; 3 Psychiatric Services of Solothurn and University of Basel, Switzerland; 4 Department of Sport, Exercise and Health, University of Basel, Basel, Switzerland; 5 Division of Substance Use Disorders, Psychiatric University Clinics Basel, Switzerland; 6 University of Zurich, Department for Psychiatry, Psychotherapy and Psychosomatics, Psychiatric Hospital, Switzerland; 7 Kermanshah University of Medical Sciences (KUMS), Kermanshah, Iran; 8 School of Medicine, Tehran University of Medical Sciences, Iran; 9 Center of Competence of Disaster Psychiatry and Disaster Psychology; Center of Competence of Disaster Medicine of the Swiss Armed Forces, Basel, Switzerland

**Keywords:** adult concentration inventory (ACI), insomnia, physical activity

## Abstract

**Background:**

Individuals with cognitive disengagement syndrome (CDS) report both lower physical activity levels and more insomnia than the general population. However, reliable data on adults with CDS are missing so far. The aims of the present study were three-fold: (1) to investigate the associations between CDS and physical activity patterns among young adults, and more specifically dimensions of physical activity (walking time/week, bicycling time/week, and aerobic physical activity/week), (2) to explore, if CDS scores, physical activity patterns, and insomnia were interrelated, and (3) to explore, if physical activity was directly or indirectly associated with CDS via decreased insomnia.

**Method:**

A total of 246 young adult students (*M*_age_ = 22.62; 56.3% females) participated in the present cross-sectional study. They completed a booklet of questionnaires covering socio-demographic information, cognitive disengagement syndrome (Adult Concentration Inventory; ACI), physical activity patterns (International Physical Activity Questionnaire; IPAQ), and insomnia (Insomnia Severity Index; ISI).

**Results:**

Higher scores for the ACI as a proxy of CDS were associated with lower physical activity patterns (durations of walking, bicycling, and aerobic exercising per week), and with higher scores for insomnia. Conditional effects modelings showed that while there was no direct and indirect association of physical activity on CDS scores, both a direct and indirect association of insomnia via lower physical activity on higher CDS scores was observed.

**Conclusions:**

Among a smaller sample of young adults, higher CDS scores were associated with lower physical activity patterns and with more insomnia. Given that standardized behavioral intervention programs are available to improve both daily and weekly physical activity patterns and insomnia, such interventions might also favorably improve CDS.

## Introduction

Young adults assume larger responsibilities as regards their social position, including mating, and their current and future vocational education, including their academic career (Sternberg, 2017). Vocational and academic success is associated with the ability to stay cognitively focused on academic tasks. By contrast, young adults suffering, for instance, from attention-deficit/hyperactivity disorder (ADHD) report more difficulties staying cognitively focused; they have lower school and academic grades, higher rates of school break-ups, more failures in vocational trainings, more social issues at the workplace, and lower incomes than their non-ADHD counterparts (Andreou & Argatzopoulou, 2024; Arnold et al., 2020; Stevens et al., 2022).

### Cognitive Disengagement Syndrome

Besides the cognitive performance-ADHD-link, in recent years, a growing body of research has focused on a similar, though clinically distinguishable cognitive behavioral set labeled cognitive disengagement syndrome (CDS; formerly: sluggish cognitive tempo [SCT]).

Cognitive disengagement syndrome (CDS) refers to a set of attentional symptoms that includes daydreaming, staring, mental fogginess/confusion, slowed behavior/thinking, and hypoactivity (Barkley, 2012; Becker et al., 2023). More specifically, factor analyses showed a two-factor model of cognitive disengagement (e.g., in a fog/confused and stares/preoccupied/in own world) and hypoactivity (e.g., sluggish/slow moving/low energy, drowsy/sleepy/not alert, and tires easily; Mayes, Becker, & Waschbusch, 2025; Mayes et al., 2023). Further, cross-sectional, longitudinal, and meta-analytic studies repeatedly report that CDS is also factor-analytically distinguished from inattentive symptoms of the attention-deficit/hyperactivity disorder (ADHD-IN; Becker, 2021) and from autism spectrum disorder (Mayes, Becker, & Waschbusch, 2025).

While the concept of CDS is now well-established in the field of child and adolescent psychopathology (Becker, 2025; Becker et al., 2023), this is less the case for adult psychopathology, despite the fact that adults seeking an ADHD evaluation also score high on CDS (Mitchell et al., 2020), and despite the fact that Barkley reported a prevalence rate of 5.1% of CDS cases in a representative sample of 1,249 U.S. adults aged 18 to 96 years (Barkley, 2012).

### Adult Symptoms of Cognitive Disengagement Syndrome (CDS) in Relation to Cognitive, Attentional, Emotional, Behavioral, and Sleep-Related Difficulties

For the CDS-cognitive performance-link, data from 60 cross-sectional and longitudinal studies showed that higher scores for CDS were associated with poorer academic performance (Fredrick & Becker, 2023), poorer skills in daily life executive functioning, greater functional impairment in specific domains of educational activities and work (see also Barkley, 2012), and with more unfavorable academic (Becker et al., 2022) and neurocognitive (Tamm et al., 2024) dimensions.

As regards the associations between CDS scores and sleep parameters, higher scores for CDS were associated with more impaired sleep patterns (Fredrick, Yeaman, et al., 2022; Sadeghi-Bahmani & Brand, 2022), and with a lower sleep quality, along with more daytime dysfunctioning (Becker et al., 2014), with more sleep disturbances, along with higher scores for symptoms of stress, depression, functional impairment (Gloger & Suhr, 2020). Among 274 outpatients aged 18 to 64 years with a broad variety of diagnosed psychiatric disorders, CDS scores showed stronger first-order and unique associations than ADHD-IN with sleep problems, along with symptoms of depression, anxiety, and stress (Yucens et al., 2024).

For the physical activity/exercising-CDS-link among adults, research is scarce, despite the fact that one of the two key factors of CDS is hypoactivity. Among 302 adolescents aged 12 to 14 years, higher CDS scores were most often independently associated with less extracurricular and physical activity involvement (Wiggs et al., 2023). To our understanding, this was the only study among adolescents with CDS, and no study focused on the physical activity-CDS-link among adults.

### Physical Activity Patterns and Sleep

Cross-sectional, longitudinal, meta-analytic, and above all interventional studies showed sufficient evidence of the physical activity-sleep-link: To illustrate, a 3-week- lasting jogging intervention in the morning improved both subjective and objective sleep parameters among adolescents (Kalak et al., 2012). Regular physical activity improved objective and subjective sleep among adults suffering from insomnia (Riedel et al., 2024), and physical activity and sleep appeared to influence each other in a bidirectional fashion (Chennaoui et al., 2015; Heiland et al., 2021; Pesonen et al., 2022). By contrast, exercising did not improve sleep quality among subjective and objective sleep among individuals with ADHD (González-Devesa et al., 2025), and for the physical activity-sleep-link among adults with CDS, it appears that such research is missing so far.

### The Present Study

Young adults are particularly under pressure to perform cognitively, emotionally, psychosocially, and as regards their academic and vocational track, though CDS interferes with demands to perform. However, the link between physical activity/exercising, sleep, and CDS has not been investigated among young adults. To counter this, we assessed a sample of Swiss university students, who completed a series of self-rating questionnaires on CDS, insomnia, and physical activity.

The following two hypotheses and study questions were formulated. First, following Wiggs et al (2023) on the research among adolescents, we expected that higher scores for CDS would be associated with lower scores for physical activity. Second, following previous studies (Becker, 2014; Fredrick, Yeaman, et al., 2022; Gloger & Suhr, 2020; Lunsford-Avery et al., 2021; Sadeghi-Bahmani & Brand, 2022; Yucens et al., 2024), we hypothesized that higher scores for CDS were associated with higher insomnia scores. The first explorative research question was, whether physical activity or insomnia or both were independently associated with CDS scores. The second research question was, whether in conditional effects modelings insomnia was associated with CDS scores directly or indirectly via physical activity, or whether physical activity was associated with CDS scores directly or indirectly via insomnia.

We believed that the present data have the potential to add to the current CDS literature in the following important ways: First, we assessed the physical activity-CDS-link for the first time among adults, and second, we investigated, if and to what extent insomnia was associated with the physical activity-CDS-link. To this end, young adult students completed a series of self-rating questionnaires covering CDS, insomnia, and physical activity.

## Methods

### Procedure

Similar to a previous study (Sadeghi-Bahmani et al., 2025), students of the university of Basel, (Basel, Switzerland) were invited to participate at the present cross-sectional and anonymous online study, which was performed with the online survey software Tivian^®^/Questback^®^. The manufacturer warrants that all data are securely stored on the manufacturer’s server, that no third parties have access to the data, and that no hidden information of a participant, such as the IP-address, will be gathered and stored.

On the first page of the online study, participants were fully informed about the aims of the study, the anonymous data gathering and data elaboration. Participants were also informed that participation or non-participation had no advantages or disadvantages for the continuation of the study, and that a participant could stop or interrupt the participation at any time. The first page of the online study further indicated that the present data might be used for scientific research and publication. Next, to “sign” the written informed consent, participants were asked to tick the following box: “I have understood the aims of the study, including the anonymous data gathering and the secure and anonymous data handling. I know that I can withdraw from the study without further consequences, and I know, who I contact in case of further study-related questions.” Afterwards, participants completed a booklet of self-rating questionnaires covering sociodemographic information, symptoms of CDS, and symptoms of psychological ill-being, that is: Depression, anxiety, stress, and insomnia. On average, participants needed about 30 to 35 minutes to complete the online questionnaires (see details below). The study lasted from November 11 to December 31st, 2024). The local ethical committee (Ethikkommission Nordwest- und Zentralschweiz (EKNZ), Basel, Switzerland) approved the study (Register-nr: Req-2024-01463; approved on 11 November 2024), which was performed according to the seventh (World Medical Association, 2013) and current version of the Declaration of Helsinki.

In a previous manuscript (Sadeghi-Bahmani et al., 2025), we investigated the associations between CDS, depression, anxiety, stress, and insomnia among this sample, though the data reported in the present manuscript are novel, in that the focus of the present manuscript was on physical activity patterns.

### Participants

Inclusion criteria were: 1. Age between 18 and 30 years; 2. Student at the University of Basel (Basel, Switzerland); 3. Willing and able to comply with the study requirements, including the intermediate to mastery level in German; 4. Ticking the box to “sign” the written informed consent. Exclusion criteria were: 1. Withdrawing from participation; 2. Pregnancy or breast-feeding, as such stages may alter both mood and sleep. 3. “Click-throughs” who needed less than 5 min to complete the questionnaires.

A total of 746 individuals read the first page, 512 (68.63%) started the online survey and 246 (32.98%) completed it. Inspection of the duration to complete the online questionnaire did not identify “click-throughs.” One participant (0.13%) self-declared to be gender-diverse; given this very low prevalence rate of self-declared individuals being divers, this data set was not further considered for data analysis. The full data set consisted of 245 participants (32.84%; mean age: 22.62 years (*SD* = 3.10); 56.3% females).

### Measures

#### Sociodemographic Information

Participants reported on their gender (male; female; or diverse) and age (years).

#### Adult Concentration Inventory (ACI)

The ACI is a self-report measure to assess Cognitive Disengagement syndrome (CDS) symptoms (Becker, Burns, et al., 2018; Fredrick, Burns, et al., 2022; Sadeghi-Bahmani et al., 2023). Typical items are: “I’m slow at doing things,” or “I get lost or drowsy during the day,” or “I get lost in my own thoughts.” Each item is rated on a four-point scale (0 = not at all, 1 = sometimes, 2 = often, and 3 = very often), with a higher sum score reflecting a more pronounced tendency toward a cognitive disengagement syndrome (CDS; Cronbach’s alpha = .94). As in previous studies (Sadeghi-Bahmani et al., 2023; Yucens et al., 2024) the 15 items version, and not the 16 items version was used, as in previous validations the item “I’m not motivated” turned out to be unrelated to CDS.

To accurately translate the English version of the ACI into German, we followed the algorithms proposed by Brislin (1986), Beaton et al. (2000), and Sousa and Rojjanasrirat (2011; see also Sadeghi-Bahmani et al., 2022, 2023): (i) two independent translators with expertise in both German and English translated the English version of the ACI into German; (ii) a third independent person with expertise in both German and English compared the two translations; (iii) where there were differences, the three experts discussed the issues and formulated the final draft; (iv) two further and new independent translators with expertise in both German and English performed the back-translation, and (v) compared the back-translated English version with the original version; (vi) the final version reflected the general agreement of all five researchers involved in this procedure (Cronbach’s alpha = .934). The German ACI has already been used in a previous study (Sadeghi-Bahmani et al., 2025), though its psychometric validation is still ongoing.

#### Physical Activity

Participants completed the European Health Interview Survey - Physical Activity Questionnaire (EHIS-PAQ; Finger et al., 2015). This questionnaire consists of eight items and asks for health–related physical activity patterns of a typical week. Outcome variables are: Walking time/week (min); bicycling time/week (min), and aerobic physical activity/week (min). The EHIS-PAQ showed good evidence for reliability and validity for the measurement of physical activity levels at work, during transportation, and health-enhancing physical activity (Baumeister et al., 2016; Cronbach’s alpha of the present study = .709).

#### Insomnia

To assess insomnia, participants completed the German version (Gerber et al., 2016) of the Insomnia Severity Index (ISI; Bastien et al., 2001). It includes seven questions about sleep quality and insomnia; participants answered how often certain conditions concerning sleep quality have occurred during the last month on scales ranging from 0 (= never/not at all) to 4 (= always). The total score ranges from 0 to 28, with a higher sum score reflecting a higher severity of insomnia (Cronbach’s alpha = .88). The following cut-off values are proposed (Bastien et al., 2001): 0 to 7 points: no insomnia; 8 to 14 points: subthreshold insomnia: 15 to 21 points: moderately clinically relevant insomnia; 22 to 28 points: severely clinically relevant insomnia. The German version of the ISI has generally acceptable psychometric properties and sufficient concurrent validity (Gerber et al., 2016; Cronbach’s alphas = .76–.81; Cronbach’s alpha of the present study = .875).

### Analytic Plan

#### Preliminary Calculations

With a series of Pearson’s correlations we investigated, if age was systematically associated with dimensions of CDS and psychological ill-being. All *r*-values were <.12 (*p* > .7). Thus, age was not introduced as a confounder.

With a series of *t*-tests we investigated if male and female participants did systematically differed in age, CDS, insomnia, and physical activity. Compared to males (*M* = 21.90; *SD* = 2.79), females were older (*M* = 23.19; *SD* = 3.22; *t*(243) = 3.30, *p* < .01, *d* = 0.43); females reported higher CDS scores (males: *M* = 8.91 (*SD* = 7.52); females: *M* = 11.40 (*SD* = 9.32; *t*(243 = 2.25, *p* < .05, *d* = 0.29) and a lower duration of weekly aerobic exercising (females: 269.57 min (*SD* = 269.56); males: 356.96 min (*SD* = 270.30; *t*(243) = 2.53, *p* = .012, *d* = 0.33). No descriptive and statistically significant mean differences were found for insomnia (males: *M* = 14.06, *SD* = 4.74; females: *M* = 15.00, *SD* = 6.12; *t*(243) = 1.30, *p* = .193, *d* = 0.17). Given the small effect sizes, the decision was to introduce Gender just as a possible confounder in the regression model.

With a series of Pearson’s correlations we explored the associations between scores for CDS and physical activity.

To explore, whether insomnia or physical activity or both were independently and more strongly associated with CDS scores, a multiple regression analysis was performed. Statistical requirements to run a multiple regression model were met (Brosius, 2018; Hair et al., 2019; Rudolf & Müller, 2004): *N* = 245 > 100; predictors explained the dependent variable (*R* = .712; *R*^2^ = .507); number of predictors: 4 (insomnia; physical activity: walking, bicycling; and aerobic PA): 10 × 4 = 40 < *N* (245); the Durbin-Watson coefficient was 1.645, indicating that the residuals of the predictors were independent. Last, the variances inflation factor (VIF) was 1.04; while there are no strict cut-off points to report the risk of multicollinearity, a VIF < 1 and a VIF > 10 indicate multicollinearity (Brosius, 2018; Hair et al., 2019; Rudolf & Müller, 2004).

Last, we followed the models described in Rudolf and Müller (2004) and in VanderWeele and Vansteelandt (2014) to test the two conditional effects models: 1. Is insomnia associated with CDS directly and indirectly via physical activity? 2. Is physical activity associated with CDS directly and indirectly via insomnia?

The level of significance was set at alpha <.05. All statistical computations were performed with SPSS^®^ 29.00 (IBM Corporation, Armonk NY, USA) for Apple Mac^®^.

## Results

### General Information

Full data were available of 245 participants. Their mean age was 22.62 years (*SD* = 3.10); 107 (43.7%) were males, and 138 (56.3%) were females.

### Associations Between Cognitive Disengagement Syndrome, Physical Activity Patterns, and Insomnia

Table 1 reports the descriptive statistical indices of CDS, indices of physical activity, and insomnia (Pearson’s correlations; *N* = 245).

**Table 1.** Descriptive Indices of and Correlation Coefficients (Pearson’s Correlations) Between Cognitive Disengagement Syndrome Scores, Indices of Physical Activity, and Insomnia (*N* = 245).

	CDS scores	Physical activity indices			Insomnia
Dimensions		Walking time/week (min)	Bicycling time/week (min)	Aerobic physical activity/week (min)	
CDS	—	−.19***	−.19**	−.25***	.70***
Walking time/week (min)		—	.41***	.50***	−.19*
Bicycling time/week (min)			—	.70***	−.12
Aerobic physical activity/week (min)				—	−.18**
*M* (*SD*); range	10.31 (8.65); 0–41	136.37 (158.70) 0–780	68.99 (110.74); 0–450	307.51 (272.84); 0–1,290	7.59 (3.57) 0–28

*Note*. CDS = cognitive disengagement syndrome.

* *p* < .05.**p < .05. ***p < .001.

Higher scores for CDS were associated with lower weekly durations (min) for walking, bicycling, and for aerobic physical activity and with higher scores for insomnia.

Longer weekly walking duration was associated with longer weekly duration for bicycling and aerobic physical activity, and with lower scores for insomnia.

Longer weekly bicycling duration was associated with longer weekly duration for aerobic physical activity.

### Regression Model to Identify Whether Insomnia or Physical Activity, or Both, Were More Robustly Associated With Scores for Cognitive Disengagement Syndrome (CDS)

Table 2 reports the regression model with cognitive disengagement syndrome scores as outcome variable, and gender, insomnia, and physical activity (weekly duration of walking, bicycling, and aerobic physical activity) as independent variables.

**Table 2.** Multiple Linear Regression With Cognitive Disengagement Syndrome Scores as Outcome Variable, and Insomnia, Gender, and Physical Activity Dimensions as Independent Variables.

Dimension	Variables	Coefficient	Standard error	Coefficient β	*t*	*p*	*R*	*R* ^2^	Durbin-Watson	VIF
CDS	Intercept	−5.866	1.481	—	3.187	<.002	.716	.513	1.904	
	Insomnia	1.053	.073	.673	14.465	<.001				1.045
	Gender	1.237	.821	.071	1.507	.133				1.057
	Walking time/week (min)	−.002	.003	−.034	−.631	.529				1.361
	Bicycling time/week (min)	−.005	.005	−.063	−.963	.337				2.049
	Aerobic physical activity/week (min)	−.002	.002	−.052	−.732	.465				2.396

*Note*. CDS = cognitive disengagement syndrome.

It turned out that higher scores for insomnia were statistically significantly associated with higher scores for CDS, while gender and all dimensions of physical activity did not reach statistical significance.

### Conditional Effects Models to Explore, If Insomnia Was Associated With CDS Directly and Indirectly Via Physical Activity, and If Physical Activity Was Associated With CDS Directly and Indirectly Via Insomnia

The correlation coefficient between CDS and insomnia was *r* = .70, and *r* = −.25 between CDS and PA (see Table 1).

Further, when only insomnia and physical activity scores were entered in the regression model, higher insomnia scores (β = .672) and lower physical activity/week scores (β = −.125) were associated with higher CDS scores.

The question arised, whether in conditional effects models higher insomnia scores were associated with higher CDS scores directly and indirectly via lower physical activity scores (Model 1), or whether lower physical activity scores were associated with higher CDS scores directly and indirectly via higher insomnia scores (Model 2). To test the models, we followed Rudolf and Müller (2004).

In sum, higher CDS scores were largely and directly associated with higher scores for insomnia, while the direct and indirect association of physical activity on CDS was modest.

## Discussion

The aims of the present study were to investigate among a sample of young adult students the associations between cognitive disengagement syndrome (CDS) scores and dimensions of physical activity and insomnia. The key findings were: (1) higher CDS scores were associated with shorter weekly physical activity durations and with (2) higher scores for insomnia. Further, (3) higher scores for insomnia were associated with higher scores for CDS both directly and indirectly via lower PA scores. By contrast, (4) the direct and indirect (via insomnia) association of physical activity on CDS was spurious. The present pattern of results adds to the current literature in the following three ways. First, this is the first study investigating the associations between CDS scores and physical activity patterns among adults. Second, we showed that higher CDS, lower physical activity, and higher insomnia were intertwined. Third, we observed that insomnia was associated with CDS directly and indirectly via physical activity, though the relative contribution of physical activity was spurious.

Two hypotheses and two research questions were formulated, and each of these is considered now in turn.

### CDS Scores and Physical Activity Patterns

With the first hypothesis, we expected that higher scores for CDS would be associated with lower physical activity patterns, and data did confirm this (see Table 1). As such, we confirmed the only study (Wiggs et al., 2023) on this topic. However, we expanded upon this previous study in the following three ways: First, it appears that the present study is the first to assess thoroughly physical activity patterns in relation to CDS among adults. Second, to assess physical activity, we used a validated and standardized self-rating questionnaire for adults, which additionally allowed the analysis of a more fine-grained physical activity pattern focusing on weekly duration of walking, bicycling, and overall aerobic exercising. Third, it appears that this is the first non-US American and non-clinical sample of young adults.

### CDS Scores and Insomnia

With the second hypothesis we expected that higher scores for CDS would be associated with higher scores for insomnia, and data did confirm this. As such, we mirrored previous findings (Becker, 2014; Fredrick, Yeaman, et al., 2022; Gloger & Suhr, 2020; Lunsford-Avery et al., 2021; Sadeghi-Bahmani & Brand, 2022; Yucens et al., 2024). The plus of the present study was that unlike the majority of previous studies, insomnia was one main outcome variable and that we used a psychometrically validated measure (ISI; Insomnia Severity Index (Bastien et al., 2001)), which allowed us to accurately report insomnia.

### CDS Scores, Physical Activity, and Insomnia

**Figure 1.**
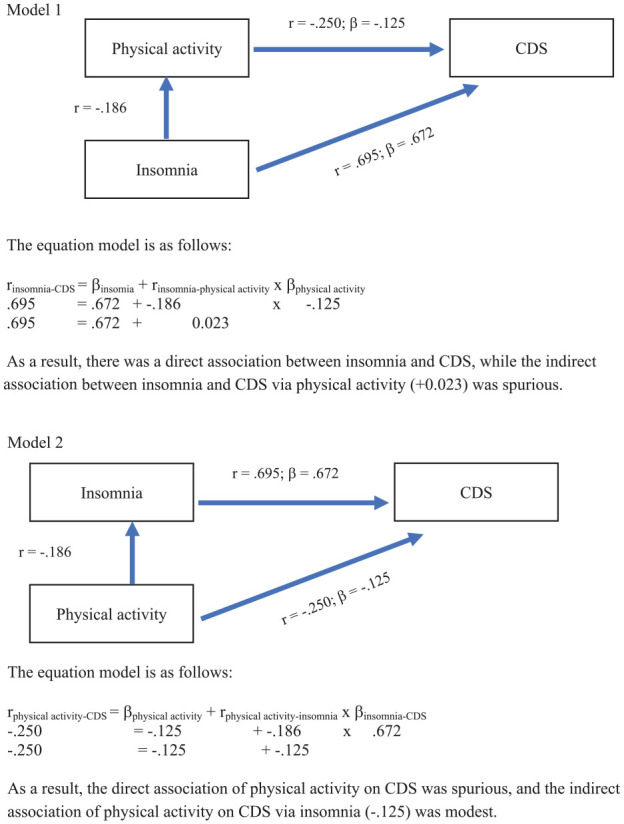
Two conditional effect models to test whether insomnia was directly and indirectly associated with CDS scores (Model 1), or whether physical activity was directly and indirectly associated with CDS scors (Model 2).

The exploratory research questions asked, whether physical activity or insomnia or both were independently associated with CDS scores, and from the regression model (Table 2), it turned out that insomnia, but not physical activity patterns, was robustly associated with higher CDS scores. Along with this, the second research question asked, whether insomnia was associated with CDS scores directly or indirectly via physical activity, or whether physical activity was associated with CDS scores directly or indirectly via insomnia. Figure 1 shows that insomnia was directly associated with CDS scores, while the indirect association via physical activity was modest. Complementarily, the association between physical activity and CDS scores was modest, both directly and indirectly via insomnia.

The question arises as to why CDS and insomnia should be associated. While the quality of the present data does not allow for understanding the precise underlying cognitive-emotional and physiological processes, we assume the following mechanisms: First, insomnia is associated with neurophysiological (Riemann et al., 2010) and cortical hyperarousal default network (Fernandez-Mendoza et al., 2016), and a prefrontal amygdala disconnect (Yoo et al., 2007). Further, in line with this, individuals with internalizing problems such as anxiety and depression report higher insomnia scores (Hertenstein et al., 2022; Palagini et al., 2022; Scott et al., 2021; Wang et al., 2019) . Next, CDS is generally associated with internalizing problems (Becker et al., 2020; Fredrick et al., 2020), including mind-wandering and rumination, though, exactly, rumination is generally identified as the main trigger and dysfunctional source of insomnia (Ballesio et al., 2021; Harvey & Payne, 2002). Thus, while the present data do not allow us to prove this assumption, it is conceivable that rumination might link CDS to insomnia via increased scores for depression.

### Limitations and Future Directions

Limitations of the study were: First, the sample consisted of highly selected participants (young adult students not older than 30 years); as such, the present results should not be generalized. Second, the cross-sectional study design does not allow for understanding if CDS scores were the cause or the result of higher insomnia and low physical activity. However, for the statistical models, we used CDS as dependent variable, though such models might yield biased results (Maxwell & Cole, 2007; Maxwell et al., 2011). Given this background, future longitudinal (e.g., see Bernad Mdel et al., 2016; Burns et al., 2025; Gaur & Pallanti, 2020; Mayes, Waschbusch, et al., 2025; Smith et al., 2020) and interventional (Adler et al., 2021; Wiggs et al., 2025) study designs should help to understand the causal directions. Fourth, it is conceivable that further latent and unassessed psychological dimensions such as ADHD (-IN), medication intake for physical and psychological issues, study exams, or daily hassles may have biased two or more dimensions in the same or opposite directions. In this view, consider that about 30% of students report psychological health issues (Becker, Holdaway, & Luebbe, 2018; Dessauvagie et al., 2022; Gloger & Suhr, 2020; Harrer et al., 2019; Li et al., 2022; Pukas et al., 2022; Rockwell & Kimel, 2023; Shan et al., 2022; Suyo-Vega et al., 2022; Quek et al., 2019; Verger et al., 2010), including medical students (Regli et al., 2024). Thus, future studies should include larger samples with broader age ranges of the general population. More specifically, a power analysis would have helped to understand if and to what extent the study might have been underpowered, such to overlook more nuanced and fine-grained patterns of associations. However, with the present sample, we could confirm the hypotheses and thoroughly answer the research questions. Last, while the pattern of associations between the ACI scores, physical activity and insomnia was in line with the expectations of previous studies and thus did confirm the hypotheses, and while the Cronbach’s alpha of the German ACI is encouraging (= .934), the German ACI has not yet been fully psychometrically assessed and analyzed. As such, the overall pattern of results should be interpreted with caution.

## Conclusions

Among a sample of young adults, higher scores for CDS were associated with lower scores for physical activity and with higher scores for insomnia. Given that validated and standardized interventions are available both to improve daily physical activity and insomnia, such interventions might also favorably influence dimensions of CDS.
